# Teachers' Personal and Collective Work-Identity Predicts Exhaustion and Work Motivation: Mediating Roles of Psychological Job Demands and Resources

**DOI:** 10.3389/fpsyg.2020.01538

**Published:** 2020-08-14

**Authors:** Ola Nordhall, Igor Knez, Fredrik Saboonchi, Johan Willander

**Affiliations:** ^1^Department of Occupational Health Sciences and Psychology, University of Gävle, Gävle, Sweden; ^2^Department of Medicine and Public Health, Swedish Red Cross University College, Huddinge, Sweden

**Keywords:** personal and collective work-identity, exhaustion, self-determined work motivation, psychological job demands, psychological job resources

## Abstract

The aim of this study was to investigate the mediating roles of teachers' psychological job demands and resources regarding personal and collective work-identity, respectively, and exhaustion and self-determined work motivation, respectively. A total of 2,905 members of a Swedish teacher's trade union received an online questionnaire by e-mail; 768 individuals answered the questionnaire and so participated in this study. The data were obtained by self-reported measures (e.g., emotional and cognitive components of work-identity, psychological job demands and resources, exhaustion and work motivation) and analyzed by mediation regression analyses. The results showed that teachers' psychological job *demands* (prosocial extra-role performance) mediated relationships between cognitive personal work-identity and emotional collective work-identity, respectively, and exhaustion. Teachers' psychological job *resources* (educational inspiration) mediated relationships between emotional personal work-identity and cognitive collective work-identity, respectively, and self-determined work motivation. Thus, teachers might be *disadvantaged* by stronger personal work-related thinking and collective work-related feeling when related to exhaustion, to some extent accounted for by psychological job demands, and they might find *advantage* in stronger personal work-related feeling and collective work-related thinking when related to work motivation, to some extent accounted for by psychological job resources.

## Introduction

Prevalence of burnout syndrome among teachers is higher than in other professional groups. However, teachers may also have access to strong work motivation and engagement in their daily educational work. These two different phenomena might be predicted by teachers' work-identity and might be explained to some extent, by different aspects of teachers' psychosocial work environment.

Thus, the aim of the present study was to investigate the mediating roles of teacher's psychological job demands and resources in relations between personal and collective work-identity, respectively, and exhaustion and self-determined work motivation, respectively.

According to Hallsten et al. (2002) 9.6% of teachers suffer from burnout syndrome, while the prevalence among other workgroups in Sweden is 6%. Teacher' daily work includes several demands and difficulties (Aloe et al., 2014). For example, large classes, pupils' complex learning needs, low social support, dysfunctional interactions with colleagues, perceived expectations to care for others in a prosocial way (Santavirta et al., 2007; Szigeti et al., 2016; Gray et al., 2017). Teachers may also be exposed to threats and even violence by students and/or their parents (De Cordova et al., 2019). Such stressful work-related situations make teachers especially vulnerable to psychological distress, such as burnout syndrome, particularly exhaustion (Maslach et al., 2001; Saboonchi et al., 2012).

Teachers, however, have also access to job resources (Demerouti et al., 2001; Klusmann et al., 2008), such as autonomy, skill variety, performance feedback, opportunities for growth (Bakker and Demerouti, 2017), and mastery of skills and experiences of educational inspiration (Vansteenkiste et al., 2007; Bakker and Demerouti, 2008; Miquelon and Vallerand, 2008; Bakker and Bal, 2010; Hultell and Gustavsson, 2011; Choochom, 2016). Job resources have been shown to be strongly and positively associated with teachers' well-being and job satisfaction, such as increased work engagement and motivation, which in turn may facilitate students' active learning and achievement (Roth et al., 2007; Klassen et al., 2012; Barbieri et al., 2019). Work engagement and motivation may be defined as “a positive, fulfilling, work-related state of mind that is characterized by vigor, dedication, and absorption” (Bakker and Demerouti, 2008, p. 209). This is in sharp contrast to the concepts of burnout and exhaustion, which are characterized by a lack of energy (Saboonchi et al., 2012). In addition, several empirical studies have reported that work-related engagement and motivation vs. burnout and exhaustion have different predictors (e.g., job resources and stability vs. demands and instability) and consequences (e.g., inspiration and active learning vs. depression and anxiety). This further indicates that work-related engagement and motivation may be opposite constructs to burnout and exhaustion (Schaufeli and Bakker, 2004; Hakanen et al., 2006, 2008; Bakker et al., 2014; Bakker and Demerouti, 2017).

An additional work-related phenomenon important for teachers' mental health and work motivation is work-identity; that is, how they define and categorize themselves by work-related individual and social attributes (Hogg and Terry, 2000; Klein, 2014; Knez, 2016). The question of, “Who am I?” at work denotes the concept of work-identity which involves emotional-and cognitive processes (Turner et al., 1987; Knez, 2016). Emotional processes are, e.g., attachment/belongingness/closeness, pride, esteem; and cognitive processes are e.g., mental time traveling, coherence, incorporation, and assimilation (Mael and Ashforth, 1992; Knez, 2014, 2016; Nordhall and Knez, 2018).

Personal and collective work identifications constitute two levels of work-identity, where stronger work-identity in general is associated with stronger work-related behaviors, norms, and attitudes (Riketta, 2005; Riketta and Van Dick, 2005; Lee et al., 2015; Nordhall and Knez, 2018), better mental health and lower psychological distress (Haslam et al., 2009; Jetten et al., 2012; Haslam, 2014; Steffens et al., 2016; Nordhall et al., 2018). However, the impact of work-identity on work-related outcomes has been reported to a large extent by researchers working within a social identity perspective, relating to the collective level of work-identity, such as work-organizations (Riketta and Van Dick, 2005; Haslam et al., 2009; Jetten et al., 2012), and by that neglecting the personal level of this phenomenon (Knez, 2016). For example, both personal and collective work-identity have recently been shown to predict employees' mental health, exhaustion, and work motivation (Nordhall and Knez, 2018; Nordhall et al., 2018). In consequence, the overarching theoretical framework of the present study is 2-fold: (1) Work-identity treated at a personal level (autobiographical memory perspective); and (2) Work-identity treated at a collective/organizational level (social identity perspective), comprising the variables of psychological job demands and resources.

Given the aims, the current study focuses on elucidating the work-related links between teachers' collective and personal work-identity, exhaustion, work motivation, and psychological job demands and resources. Knowledge concerning these relationships is of importance to teachers, given their work-related conditions (Barbieri et al., 2019; Parrello et al., 2019). As far as we know, no previous research has addressed these types of associations, which are of relevance to organizational psychology.

### Teachers' Job Demands and Resources

The Job Demands-Resources (JD-R) model describes how work-related environmental characteristics, in terms of job demands and resources, are related to each other and how they are related to adverse mental health, foremost burnout and exhaustion as well as work engagement (Demerouti et al., 2001; Bakker and Demerouti, 2017; Hakanen and Bakker, 2017). Teachers' job demands have been shown additionally to positively associate with energetic processes (e.g., wearing out and exhausting), and job resources have been shown to positively associate with motivational processes (e.g., engagement and commitment). By this, teachers' job demands and resources account to some extent for teachers' work-related well-being (Hakanen et al., 2006).

Concerning teachers' work setting, the JD-R model asserts that teachers' job resources may reduce the impact of job demands on outcomes such as exhaustion (Hakanen et al., 2006; Bakker and Demerouti, 2008; Demerouti and Bakker, 2011). For example, when entering employment, teachers may develop burnout as a result of job demands, e.g., unmet expectations from parents and pupils, but increase their work engagement as a result of job resources, e.g., mastery of teaching skills (Hultell and Gustavsson, 2011). In addition, it has been shown that teachers' job resources such as autonomy and opportunities for development in teaching may positively relate to work engagement (Hakanen et al., 2006; Bakker and Bal, 2010).

The *energetic* process relates to job *demands* defined as those physical, psychological, social or organizational aspects of the job that require sustained physical and/or psychological effort and therefore are associated with some physiological or psychological costs (Demerouti et al., 2001; Bakker and Demerouti, 2017). One such *differential* demand, which may explain individual differences in teachers' psychological functioning (Klusmann et al., 2008), is emotionally challenging social interactions. For example, prosocial extra-role performance implying perceived expectations to care for others, e.g., students, colleagues and pupils' parents, in a prosocial way (Santavirta et al., 2007; Szigeti et al., 2016; Gray et al., 2017). Increased prosocial extra-role performance may be associated with better mental health (Simbula and Guglielmi, 2013; Lam et al., 2016). However, other findings have indicated a “dark side” of this phenomenon in that burnout and exhaustion may increase as a consequence of stronger prosocial extra-role performance (Bolino et al., 2004, 2013; Bolino and Turnley, 2005; Vigoda-Gadot, 2006; 2007; Bolino and Grant, 2016). It has been suggested that burnout, as the main manifestation of a work-related adverse psychological condition, comprises the dimensions of exhaustion, depersonalization, and reduced efficacy (Maslach and Jackson, 1981; Maslach et al., 2001; Maslach and Leiter, 2008). Most of the current definitions and measurement models of burnout include the concept of e*xhaustion* (Saboonchi et al., 2012; Grossi et al., 2015).

The conceptualizations and assessments of burnout varies considerably, including a recent approach to the phenomenon as a process rather than a state (Hallsten, 2017). However, exhaustion taps the health status and symptom presentation in individuals exposed to prolonged stress (see Schaufeli et al., 2017 for a review). As such a state, it appears more readily assessable. Exhaustion can be described as a lack of energy in social interactions, physical fatigue, inability to accomplish/cope with everyday demands, impaired memory, concentration difficulties, sleeping problems, and emotional instability (Saboonchi et al., 2012). Furthermore, it has been shown that exhaustion increases with higher job demands and that decreasing psychological demands results in lower levels of exhaustion (van der Ploeg and Kleber, 2003; Janssen and Nijhuis, 2004; Chrisopoulos et al., 2010; Van de Ven et al., 2013).

The *motivational* process relates to job *resources* defined as physical, psychological, social or organizational aspects of the job. These aspects might involve, e.g., autonomy, skill variety, performance feedback and opportunities for growth, that are functional in achieving goals, personal growth, development and learning and reducing costs associated with job demands (Demerouti et al., 2001; Bakker and Demerouti, 2017). Stronger job resources associate with stronger work-related motivation, engagement, commitment (Hakanen et al., 2006, 2008; Bakker and Demerouti, 2017). According to Self-Determination Theory, teachers' intrinsic work motivation is based on pure interest, enjoyment and inner inspiration and is self-determined (Ryan and Deci, 2000a, 2017; Deci and Ryan, 2002; Corkin et al., 2018). This contrasts with non-self-determined motivation based on external contingencies like rewards. Therefore, psychological needs of autonomy, relatedness and competence might be promoted by teachers' occupational work *per se* and these psychological needs may thus influence self-determined motivation at work (Ryan and Deci, 2000b; Hakanen et al., 2006; Deci and Ryan, 2009; Van den Broeck et al., 2016).

Teachers' experiences of educational inspiration involves psychological aspects of the job situation facilitating a strong dedication and psychological engagement in one's work (see Ryan and Deci, 2000a; Mauno et al., 2007; Bakker and Demerouti, 2008; Bakker and Bal, 2010). Such experiences may be related to internal regulatory processes positively associating with self-determined motivation at work. By educational inspiration teachers may also experience personal growth, learning and development, such as stimulating educational interactions with the students and overall incentive educational work (see Ryan and Deci, 2000b; Deci and Ryan, 2002; Gagne and Deci, 2005; Hakanen et al., 2006; Klusmann et al., 2008).

Educational inspiration may positively predict, but is not equivalent to, intrinsic work motivation; that is “by satisfying the basic psychological needs of autonomy, belongingness and competence, job resources are also intrinsically motivating for employees” (Hakanen et al., 2008, p. 225). Although tapping the concept of personal resources, e.g., optimism and personal values, educational inspiration may constitute a psychological *job* resource (see Bakker and Demerouti, 2008; Klusmann et al., 2008; Barni et al., 2019). Here educational inspiration might be situated in a well-defined working context of some teachers, as an experiential aspect of the job, and as such may buffer against negative effects of job demands (see Vansteenkiste et al., 2007; Bakker and Demerouti, 2008; Miquelon and Vallerand, 2008; Bakker and Bal, 2010; Choochom, 2016). Intrinsic goals, like inspiration in one's work, have been associated with self-determined motivation and vitality at work. Accordingly, inspiration in one's work may generate self-determined motivation because it satisfies the basic psychological needs of autonomy, relatedness and competence (Hakanen et al., 2008; Ryan and Deci, 2008; Van Den Broeck et al., 2008). By this, such inspiration may increase job satisfaction and well-being (Ryan and Deci, 2000b; Björklund et al., 2013).

In view of this, teachers' work-identity might be one important factor predicting experiences of psychological job demands and resources.

### Teachers' Work-Identity

Another important concept related to teachers' work motivation and mental health is work-identity (Nordhall and Knez, 2018; Nordhall et al., 2018). Here, individuals categorize and define themselves in terms of individual attributes, encompassing personal self/identity (Hogg and Terry, 2000; Klein, 2014; Knez, 2016; Knez et al., 2019). However, individuals also categorize and define themselves in terms of social attributes, involving collective self/identity (Jackson et al., 2006; Knez, 2016).

Work-identity includes two levels of identity tapping separate knowledge structures (Kihlstrom et al., 2003). The *personal* level means *teacher* as a profession (see Kremer and Hofman, 1985; Fisherman, 2015; Knez, 2016) and the *collective* level, *school* as an organization (see Van Dick and Wagner, 2002; Riketta, 2005; Pate et al., 2009; Miscenko and Day, 2016). Thus, teachers' work-identity includes an *individual* level, referring to personal autobiographical work-related emotions and cognitions (Knez, 2014, 2016; Knez et al., 2019). Accordingly, teachers' work identity also includes a *social* level relating to collective work-related associations and aspirations, and applies at multiple levels of abstraction (Pate et al., 2009; Knez, 2016).

In view of this, teachers' personal work-identity implies categorizations of *I/Me*, as a teacher encompassing the fundamental human need of *distinguishing* I/me from others (Brewer and Gardner, 1996) “in order to preserve the personal self, the personal story and its memories” (Knez, 2016, p. 3). Accordingly, personal work-identity is related to personal self-related behaviors, motivations, attitudes, values, interests and cognitions in that stronger personal work-identity means stronger work-related personal goals, preferences and needs (Brewer and Gardner, 1996; Ybarra and Trafimow, 1998; Ellemers et al., 2004; Johnson et al., 2006).

On the other hand, teachers' collective work-identity involves *We*-descriptions (Knez, 2016), *We* working at the XX school. This is consistent with our need to *belong* to a social group (Brewer and Gardner, 1996) “in order to be part of the collective self, the collective story and its memories” (Knez, 2016, p. 3). Consequently, collective work-identity incorporates behaviors, motivations, attitudes, values, interests and cognitions related to the collective (Ybarra and Trafimow, 1998; Johnson et al., 2006). In accord with social identity theory (Tajfel and Turner, 1979, 1986; see Hogg, 2012 for a review), stronger collective work-identity suggests a depersonalization of the individual self, resulting in stronger psychological bonding to the work-group/organization (Hogg and Terry, 2000; Ellemers et al., 2004; Johnson and Jackson, 2009).

Given the above, both emotion and cognition components have been shown to account for effects associated with personal and collective work-identity.

### Emotion and Cognition in Teachers' Work-Identity

A conceptual model for work-related self/identity, encompassing *emotional* and *cognitive* processes accounting for the phenomenon of work-bonding has been suggested by (Knez, 2014, 1996, see also Van Dick and Wagner, 2002). The emotion component includes the process of attachment/belongingness/closeness to the work, and the cognition component involves work-related processes of coherence, correspondence, mental time, reflection, and agency (see also Conway et al., 2004; Klein et al., 2004). The formation of the emotion component is suggested to precede the formation of the cognitive one when establishing work-identity (Knez, 2016, see also Knez and Eliasson, 2017; Knez et al., 2018). The (Brewer and Gardner, 1996) model is based on autobiographical memory accounts and thus is general in that it proposes basic psychological processes accounting for both personal- and collective types of person-work bonding (see Conway et al., 2004; Klein et al., 2004; Knez, 2014, Knez, 2017; Knez and Nordhall, 2017; Knez et al., 2017). Thus, the (Brewer and Gardner, 1996) is also in line with the definition of social identity suggested by Tajfel (1972) involving emotional and cognitive processes in identity formation (see Tajfel, 1978; Haslam and Ellemers, 2011; Hogg, 2012).

Collective work-identity, described by (Corley et al., 2006 p. 88) as “a product of the dialectic relationship between collective, shared cognition on the one hand and socially structured individual cognitions on the other,” is supposed to be more of a cognitive entity and is a contrast to personal work-identity (Ashforth and Mael, 1989; Harquail and King, 2003; Knez, 2016).

Finally, the cognitive component of collective work-identity (in terms of incorporation, identification and assimilation) is suggested to precede the emotional component (in terms of pride, esteem, and affective commitment) when collective work-identity, is accountable for psychological wellness at work (see Mael and Ashforth, 1992; Keyes, 2005; Van Knippenberg and Sleebos, 2006; Howard et al., 2016; Van den Broeck et al., 2016; Nordhall and Knez, 2018; Nordhall et al., 2018).

Given teachers' emotional and cognitive work-identity in relation to their exhaustion problems and work motivation it is relevant to understand the role of psychological job demands and resources in these relationsships.

### The Present Study

Work-related outcomes, such as motivation and justice at work, may link differently to the emotion- and cognition components of personal and collective work-identity (Nordhall and Knez, 2018). In addition, Nordhall et al. (2018) showed that when cognitive processes increase in personal work-identity and when emotional processes increase in the collective work-identity, teachers report a lower general mental health and, consequently, higher levels of exhaustion (see Saboonchi et al., 2012; Nordhall et al., 2018). Thus, when teachers *think more* regarding their *personal* work-identification and when they *feel* more regarding their *collective* work-identification, they feel mentally worse and are more exhausted. Similar findings have been reported previously by, for example, Kremer and Hofman (1985), Van Dick and Wagner (2002), and Fisherman (2015) regarding personal work-identity and by Cohen (2004), Cruwys et al. (2015), Greenaway et al. (2015a,b), and Jetten et al. (2014) concerning collective work-identity.

Based on previous research, it might be suggested that the *cognitive* component of the *personal* work-identity and the *emotional* component of the *collective* work-identity are *positively* associated with work-related psychological distress, e.g., exhaustion (e.g., Kremer and Hofman, 1985; Van Dick and Wagner, 2002; Jetten et al., 2014; Fisherman, 2015; Nordhall et al., 2018). Practically, this suggests that teachers *thinking* in their self-work bonding and *feeling in* their self-organization bonding may partially account for their work-related psychological distress.

Furthermore, it has been reported that the positive association between personal work-identity and self-determined motivation at work (Ryan and Deci, 2000b; Gagne and Deci, 2005; Tremblay et al., 2009) is accounted for by the *emotion* component. Thus, when the emotional processes in personal work-identity increase, work self-determined motivation also increases (Nordhall and Knez, 2018).

Regarding the collective work-identity, more “collectivized” individuals will manifest stronger acceptance, loyalty, adherence to decisions, beliefs, values and norms, communicated by the collective (Hogg and Terry, 2000; Ellemers et al., 2004; Johnson and Jackson, 2009). With regard to work motivation, this suggests that a depersonalized individual, by the cognitive processes of incorporation, identification and assimilation in their collective work-identity, may convert individual motivational and normative mechanisms into *We*-descriptions (see Mael and Ashforth, 1992; Van Knippenberg and Sleebos, 2006; Nordhall and Knez, 2018). Thus, the variance in the self-determined motivation at work will be positively accounted for by the cognitive vs. the emotional component when collective work-identity is involved (Brewer and Gardner, 1996; Ybarra and Trafimow, 1998; Ellemers et al., 2004). Consequently, a positive association between collective work-identity and work-related motivation is stronger in a collectivistic vs. an individualistic organizational culture (Lee et al., 2015). Accordingly, when personal work-identity is dominant in the work motivation processes, it is suggested that the emotion component of work-identity is primarily accountable, and the reverse when collective work-identity, primarily governing the cognitive component, is responsible for the work motivation processes.

This suggests that, when teachers *feel more* in their *personal* work bonding, and *think more* in their *collective* work bonding they are more self-determined in their motivation at work (see Nordhall and Knez, 2018).

Individual differences in social and personal work-identity have been shown to predict burnout and exhaustion among teachers (see Nordhall et al., 2018 for an overview, see Kremer and Hofman, 1985; Van Dick and Wagner, 2002; Alarcon et al., 2009; Haslam et al., 2009; Jetten et al., 2012, 2014; Haslam, 2014; Fisherman, 2015; Steffens et al., 2016; Parrello et al., 2019 for more details). However, situational and organizational factors in terms of job characteristics have been shown to play an even more crucial role for these outcomes (Maslach et al., 2001; Angerer, 2003; De Lange et al., 2004; Schaufeli et al., 2009; Seidler et al., 2014; Badawy, 2015; Parrello et al., 2019).

One such important organizational factor is psychological job demands, which have been shown to associate positively with burnout and exhaustion. This indicates that psychological job demands may associate with psychological distress (Demerouti et al., 2001; Hakanen et al., 2006; Michielsen et al., 2007; Santavirta et al., 2007; Klusmann et al., 2008; Hultell and Gustavsson, 2011; Hultell et al., 2013; Bakker and Demerouti, 2017). It has also been suggested that obsessive work passion, such as intense rumination, repetitive and unintentional perseverative thoughts about one's work, might trigger stress-related syndromes of exhaustion (Demerouti et al., 2001; Michielsen et al., 2007; Demerouti and Bakker, 2011; Donahue et al., 2012).

As previously mentioned, teachers' working conditions and job characteristics, such as psychological job resources, have shown implications for teachers' well-being in terms of increased work engagement and motivation (Hakanen et al., 2006; De Cordova et al., 2019).

Also, Karatepe (2015) showed that personal resources, such as intrinsic values and self-efficacy may negatively mediate the negative relationship between perceived organizational support and exhaustion, and turnover intentions. This indicates that job resources are important regarding the employee and his/her mental health and work motivation and engagement (Hakanen et al., 2008).

**Figure 1:**
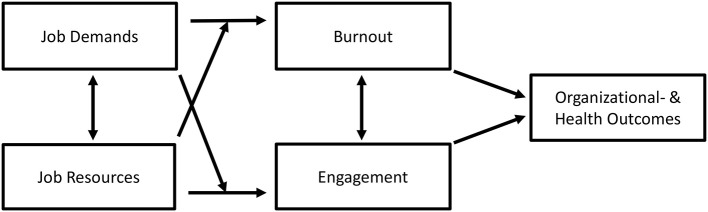
A general Job Demands-Resources model, including Job Demands, Job Resources, Burnout, Engagement, and Organizational- and Health outcomes.

The JD-R model in general emphasizes job demands and resources as predictors of burnout and work engagement, respectively, which in turn may affect organizational- and health related outcomes (see Hakanen et al., 2006, 2008; Bakker and Demerouti, 2008; Demerouti and Bakker, 2011), see Figure 1 for a general JD-R model.

However, in the present study, personal and social identity perspectives are added to the JD-R Model in that we analyze the mediating roles of psychological job demands and resources within two theoretical frameworks; (1) personal (autobiographical) memory perspective and (2) social identity perspective. By this one may gain some more empirical and theoretical knowledge of job demands and resources as mediating factors between the individual teacher (work-identity) and adverse mental health (exhaustion) and work motivation (self-determined motivation at work). To our knowledge, similar modifications have only been carried out to a lesser extent in previous research on the JD-R Model (see Bakker and Demerouti, 2017; Kaltenbrunner et al., 2019).

**Figure 2:**
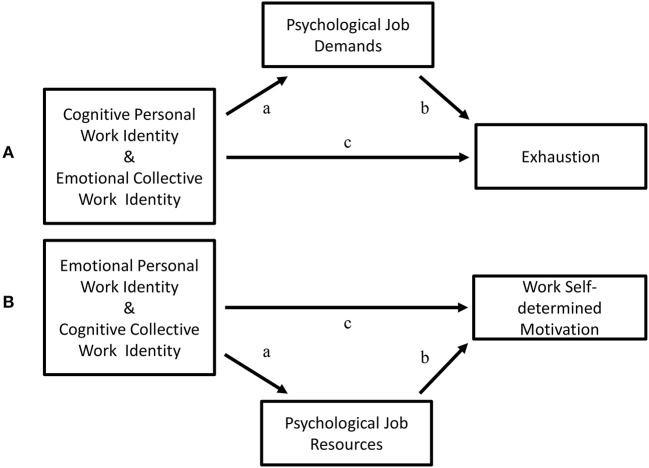
**(A)** Cognitive personal work-identity and emotional collective work identity as predictors of exhaustion = path c, mediated by psychological job demands = path ab. **(B)** Emotional personal work-identity and cognitive collective work identity as predictors of work self-determined motivation = path c, mediated by psychological job resources = path ab.

Along the lines of the above, our first aim was to investigate if teachers' psychological job demands mediated a positive relationship between their cognitive personal work-identity and emotional collective work-identity, respectively, and exhaustion (see Figure 2A showing teachers' cognitive personal work-identity and emotional collective work-identity, respectively, predicting exhaustion indirectly via psychological job demands). Given that the phenomenon of exhaustion is fundamental in definitions and measures of burnout (Saboonchi et al., 2012; Grossi et al., 2015), and that it has been incorporated in the Swedish version of the International Classification of Diseases by the Swedish Board of Health and Welfare (Socialstyrelsen, 2003, 2010), in the following we will use the concept of exhaustion instead of burnout. Although the main criteria of diagnosis for exhaustion disorder partly correspond with descriptions of a clinical level of burnout these concepts should not be treated as interchangeable (Grossi et al., 2015; Nordhall et al., 2018).

Given the above, our first hypothesis predicts that a positive relationship between teachers' cognitive personal work-identity (H1a) and emotional collective work-identity (H1b), respectively, and exhaustion is mediated by teachers' psychological job demands.

The second aim was to investigate if teachers' psychological job resources mediated a positive relationship between their emotional personal work-identity and cognitive collective work-identity, respectively, and self-determined motivation at work (see Figure 2B showing teachers' emotional personal work-identity and cognitive collective work-identity, respectively, predicting self-determined motivation at work indirectly via psychological job resources).

Given this, our second hypothesis predicts that a positive relationship between teachers' emotional personal work-identity (H2a) and cognitive collective work-identity (H2b), respectively, and self-determined motivation at work is mediated by teachers' psychological job resources.

## Method

Since the present study is a part of a larger research project, resulting in several publications on work-identity and health, the method section in general terms complies with previous publications within this project (Nordhall and Knez, 2018; Nordhall et al., 2018).

### Participants

Two thousand nine hundred and five members of the Swedish trade union, “The National Union of Teachers” (in Swedish “Lärarnas Riksförbund”), originating from 11 different local union branches and working in the south and middle part of Sweden, received an online questionnaire by e-mail. The response rate was 26% (returned questionnaires *n* = 768). Mean age of the participants was *M* = 46.3 years (*SD* = 10.07, range 24–67) and mean employment time within the organization (school) 14 years (*SD* = 10.2). Of the *n* = 768 respondents, 99% worked as teachers or similar, i.e., had an educational function, 95.2% were in permanent employment, 92.1% of the participants worked within the municipal sector, 80.5% had full-time jobs, 75.5% were female and 68% had university studies as their highest level of education.

### Procedure

We asked the chairpersons of 11 municipal associations of the Swedish trade union, “The National Union of Teachers,” to invite their union members to participate in a survey about work-identity and health. This was done because chairpersons are not, due to Swedish juridical restrictions, permitted to publish e-mail addresses of individual members outside the union, and thus the chairpersons distributed a web-link to the questionnaire to the members. A covering letter accompanied the questionnaires, describing the purpose of the project, informed the participants that completion of the questionnaire indicated their consent to participate voluntarily in the present project, and that confidentiality and anonymity were assured. The participants were asked to fill in their names and address after completing the questionnaire if they wanted to receive a cinema ticket as compensation for their participation. They were informed that only the researchers of the present study would have access to their names and addresses. In the present study, data related to emotion and cognition component of personal and collective work-identity, educational inspiration, prosocial extra-role performance, work self-determined motivation and exhaustion were analyzed.

Finally, an ethical application was reviewed and approved by the Swedish regional ethical committee of Uppsala.

### The Questionnaire

The questionnaire consisted of questions concerning background, exhaustion, work motivation, work-identity, psychological job demand and resources. The included measures are detailed below.

### Measures

#### Background Variables

Age, gender, employment time within the organization (school), type of employment, permanent vs. non-permanent employment, private vs. non-private sector, full-time vs. non-full-time job and level of education were measured as demographical background variables.

#### Exhaustion

Exhaustion was measured using the “Karolinska Exhaustion Scale 26” (KES26), developed by Saboonchi et al. (2012) which measures self-reported exhaustion problems. It includes six subscales (cognitive exhaustion, disturbed sleep, excessive fatigue, somatic symptoms, irritability, and negative affect) measured by 26 statements and the question: “How often have you experienced problems with any of the following issues during the past month?”. Responses were made on a five-point Likert scale, defined as: 1 = “never”; 2 = “seldom”; 3 = “sometimes”; 4 = “often”; and 5 = “always.” Adequate fit and good psychometric properties have been demonstrated for the one-factor model of KES 26. All the subscales, except for the subscale of disturbed sleep (consisting of only two items), have shown acceptable internal consistency: primary sample mean α = 0.76 and cross-validation sample mean α = 0.82 (Saboonchi et al., 2012). In the present study, the one factor model of exhaustion showed a Cronbach alpha value (α) of 0.95, indicating very good internal consistency (see DeVellis, 2003).

#### Work Motivation

To measure work motivation, we used the “Work Extrinsic and Intrinsic Motivation Scale,” WEIMS (Tremblay et al., 2009) which measures self-determined work motivation. WEIMS has been used in several previous studies (Peklar and Bostjancic, 2012; Stoeber et al., 2013; Jayaweera, 2015, Saltson and Nsiah, 2015). Grounded in self-determination theory (Ryan and Deci, 2000b; Deci and Ryan, 2002; Gagne and Deci, 2005), WEIMS is an 18-item measure. Participants responded to the WEIMS-items on a 5-point Likert scale, ranging from 1 (“Does not correspond at all”) to 5 (“Corresponds exactly”). Six subscales are included in WEIMS, measuring: (a) Intrinsic motivation; (b) Integrated regulation; (c) Identified regulation; (d) Introjected regulation; (e) External regulation; and (f) Amotivation. The subscales a–f were aggregated into an overall “Work self-determined index”, W-SDI (see Tremblay et al., 2009 for details) in order to obtain a measure of the participants' overall work motivation. The response scale has a range of ± 24. Self-determined work motivation is indicated by the positive part of the scale, while the negative one indicates non-self-determined work motivation (Vallerand and Ratelle, 2002; Tremblay et al., 2009). Results show good psychometric properties for this measure, with a Cronbach alpha (α) of 0.84 (Tremblay et al., 2009). The Cronbach alpha (α) of W-SDI was 0.74 in the present study, indicating acceptable internal consistency (see DeVellis, 2003).

#### Work-Identity

Work identity was measured in terms of two constructs: personal and collective work-identity. Personal work-identity was measured by an instrument developed by (Knez, 2016; see also Knez and Eliasson, 2017). The instrument comprised ten statements measuring emotional and cognitive components of personal work-identity: Emotional (“I know my work very well.”; I miss it when I'm not there.”; “I have strong ties to my work.”; “I am proud of my work.”; “It is a part of me.”); Cognitive (“I have had a personal relation with my work over a long period.”; “There is a link between my work and my current life.”; “I can travel back and forth in time mentally to my work when I think about it.”; “I can reflect on the memories of my work”; “My thoughts and memories about my work are part of me.”). The participants responded to the statements on a five-point Likert scale, ranging from 1 (completely disagree) to 5 (completely agree). The Cronbach alpha (α) value was 0.86 for personal work-identity, 0.75 for emotional- and 0.84 for cognitive component, respectively, in the present study, showing acceptable-good internal consistency (see DeVellis, 2003). Construct validity statistics for the personal work-identity construct/measure have been reported by Nordhall and Knez (2018), showing an acceptable data fit of Chi^2^ = 188.57, df = 28 (*p* = 0.000), CFI = 0.95 and RMSEA = 0.08 (see Byrne, 2016).

In order to measure collective work-identity the “Identification with a Psychological Group Scale” (Mael and Ashforth, 1992; Mael and Tetrick, 1992; Riketta, 2005), theoretically grounded in Social Identity Theory (Tajfel and Turner, 1979, 1986) and the Self-Categorization Theory (Hogg and Terry, 2000) was used. Six statements with a five-point Likert scale, ranging from 1 (completely disagree) to 5 (completely agree) are included in this measure. In line with the conceptual model of Knez (2014, 2016) that distinguishes between emotional- and cognitive components of work-identity (Jackson et al., 2006; Knez, 2016), three items of the “Identification with a Psychological Group Scale” (Mael and Ashforth, 1992) were categorized as measuring the cognitive component (“I am very interested in what others think about the organization.”; “When I talk about this organization, I usually say 'we‘ rather than 'they‘.”; “This organization's successes are my successes.”) and emotional component; “When someone criticizes my organization, it feels like a personal insult.”; “When someone praises the organization it feels like a personal compliment.”; “If a story in the media criticized the organization, I would feel embarrassed.”), respectively. This was done in line with Mael and Ashforth, 1992 suggestions (also supported by Tajfel, 1972, 1978; Hogg, 2012). The different scales showed the following Cronbach alphas (α):0.87 for collective work-identity,0.78 for emotional- and 0.77 for cognitive component, indicating good internal consistency (see DeVellis, 2003). In addition, and as above, Nordhall and Knez (2018) reported an acceptable construct validity data fit of Chi^2^ = 64.09, df = 7 (*p* = 0.000), CFI = 0.97, and RMSEA = 0.10 for the collective work-identity concept/measure (see Byrne, 2016).

Job demands and resources. Psychological job demands were measured by three items, assessing teachers' prosocial extra-role performance (“extra-role support given to the pupils; colleagues; pupils' parents,” respectively), on a five-point Likert-scale ranging from 1 (completely disagree) to 5 (completely agree). Cronbach alpha (α) for this scale was 0.64, indicating moderate consistency (see DeVellis, 2003).

Psychological job resources were measured by two items assessing educational inspiration (“stimulating educational interactions with the students”; “overall educational work as incentive”) on a five-point Likert-scale ranging from 1 (completely disagree) to 5 (completely agree). Cronbach alpha (α) for this scale was 0.73, indicating acceptable consistency (see DeVellis, 2003).

The items measuring psychological job demands and resources were developed by the authors of the present study and based on the Job Demands-Resources model (see Demerouti et al., 2001).

### Design and Data Analyses

In accordance with the two hypotheses (see Introduction), four types of mediation analyses were performed by Hayes (2018) PROCESS (Hayes et al., 2017) macro for SPSS (version 2.16.3), model 4. The mediation, i.e., the indirect effect of IV on DV was defined as the product of path a and b, i.e., ab (see Figures 1, 2). In most situations, ab = c – c', where the total effect of IV and DV, known as path c (see Figures 1, 2), can be decomposed into a direct component: path c', and an indirect component: path ab (MacKinnon et al., 2000, 2007; Preacher and Hayes, 2004). A bias-corrected (BC) 5,000 bootstrapped confidence interval (CI) was generated by PROCESS 2.16.3 identifying the upper and lower bounds of a 95% CI for the indirect effect (see Hayes, 2018). The indirect effect was regarded as statistically significant when zero was not included between the lower limit of the 95% confidence interval (LLCI) and the upper limit of the 95% confidence interval (ULCI), independent of the total effect of X on Y. Although not in accordance with the definition of mediation in the original work by Baron and Kenny (1986), the definition adopted in the present paper is more in line with current definitions of mediation (see MacKinnon, 2008; Hayes, 2009; Zhao et al., 2010; Hayes and Rockwood, 2016).

The relationships between the predictors and the outcome variables in the present data set have been shown *not* to be affected by: monthly income; school sector (public vs. private); years of employment; and permanent employment (no vs. yes) (Nordhall and Knez, 2018); age; gender (male vs. female); part- or full-time employment (%); school sector (public vs. private); years of employment; and educational level (low vs. high) (Nordhall et al., 2018). Accordingly, we have not included these variables in the present analyses.

Due to the study's ethical considerations we could not collect any data on teachers' school affiliations. Therefore, no intra-class-correlations (ICC) were calculated.

## Results

First, we report the bivariate correlations, *N*, mean and standard deviation statistics for all variables included in the regression analyses (see Table 1). Second, we report the results obtained in accordance with our hypotheses and the types of mediation analyses associated with each one of the two hypotheses (H1a-b; H2a-b), respectively.

**Table 1:** Bivariate correlations (r), *N*, mean (*M*), and standard deviation (*SD*) statistics for all variables included in the mediation analyses: Emotional (E-) and cognitive (C-) component of personal work-identity (PWI); emotional (E-) and cognitive (C-) component of collective work-identity (CWI); exhaustion (E); work self-determined motivation (WSDM); psychological job demands (JD); and psychological job resources (JR).

	**N**	**M**	**SD**	**E-PWI**	**C-PWI**	**E-CWI**	**C-CWI**	**EE**	**WSDM**	**JD**
E-PWI	767	3.62	0.71							
C-PWI	767	3.61	0.86	0.61^**^						
E-CWI	767	2.59	0.99	0.25^**^	0.23^**^					
C-CWI	767	2.97	0.97	0.29^**^	0.22^**^	0.74^**^				
E	767	2.43	0.74	−0.13^**^	0.15^**^	0.01	−0.09*			
WSDM	766	4.97	6.05	0.59^**^	0.32^**^	0.12^**^	0.22^**^	−0.33^**^		
JD	766	2.68	0.79	0.06	0.13^**^	0.12^**^	0.09*	0.33^**^	−0.11^**^	
JR	766	4.51	0.63	0.48^**^	0.21^**^	0.11^**^	0.19^**^	−0.18^**^	0.44^**^	0.07

** Correlation is significant at the 0.01 level (2-tailed). *Correlation is significant at the 0.05 level (2-tailed).

**Figure 3:**
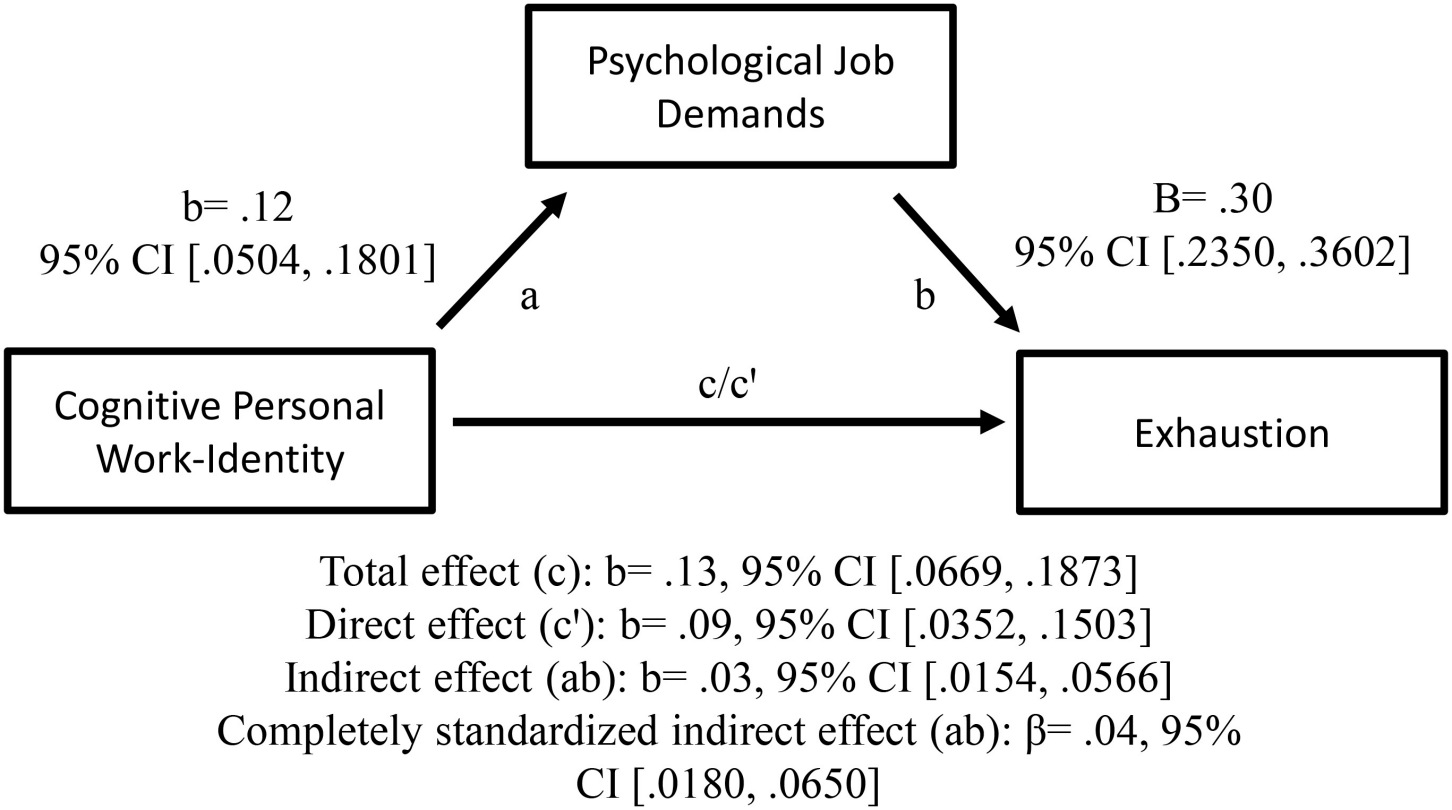
Cognitive personal work-identity as a predictor of exhaustion, mediated by psychological job demands. The confidence interval (CI) for the indirect- and completely standardized indirect effect is a BC bootstrapped CI based on 5,000 samples.

**Figure 4:**
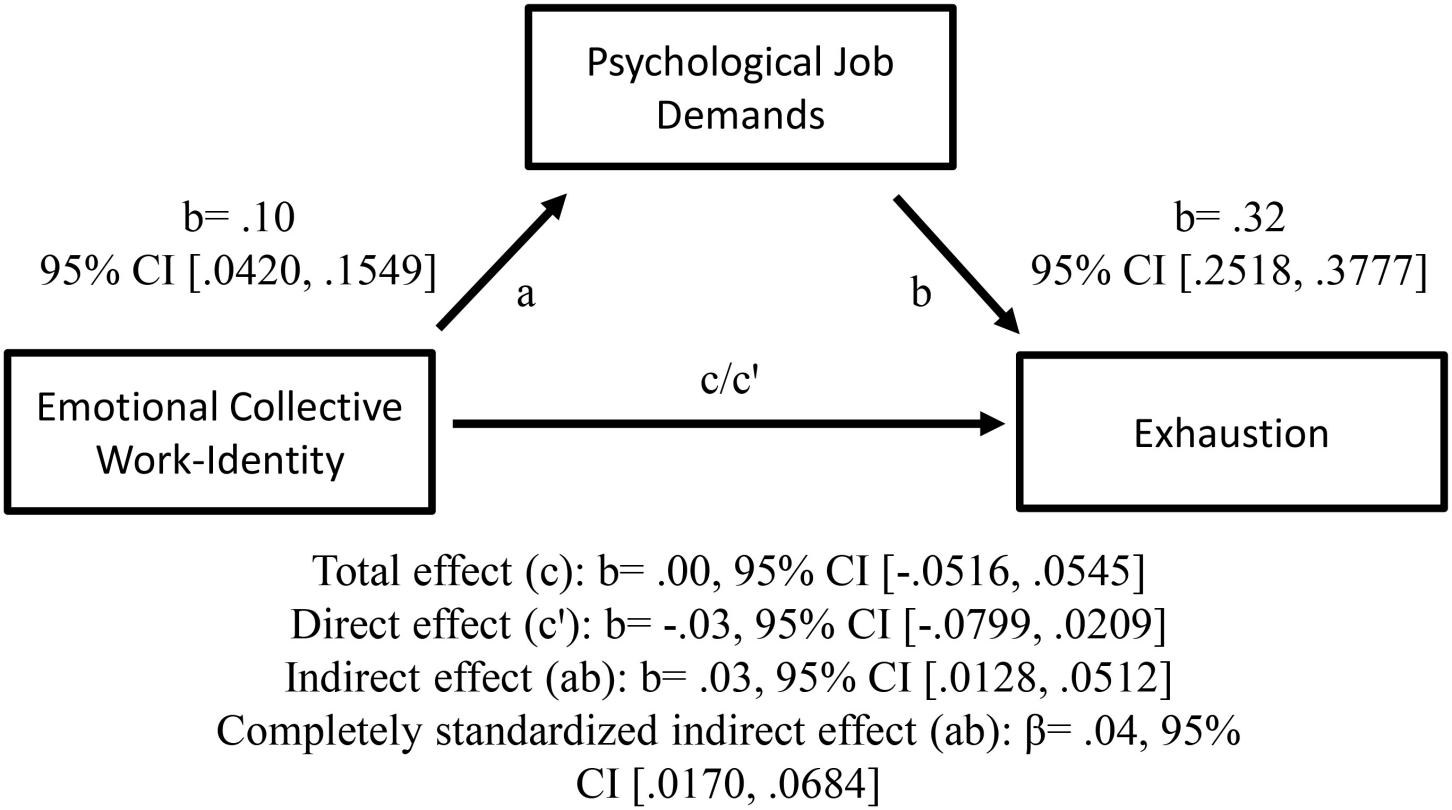
Emotional collective work-identity as a predictor of exhaustion, mediated by psychological job demands. The confidence interval (CI) for the indirect—and completely standardized indirect effect is a BC bootstrapped CI based on 5,000 samples.

As predicted, the positive relationships between teachers' cognitive personal work-identity (H1a) and emotional collective work-identity (H1b), respectively, and exhaustion, were mediated by psychological job demands (in terms of prosocial extra-role performances); see Figures 3, 4 for details. Thus, teachers' cognitive personal work-identity and emotional collective work-identity, respectively, predicted exhaustion indirectly via psychological job demands. As can be seen in Figure 4, the total effect was not significant and the different signs of the direct (c'), and the indirect effect, might indicate an inconsistent mediation (MacKinnon et al., 2000). However, since (1) the total effect (c) just equals the sum of direct (c') and indirect (a × b) effects, and (2) the indirect effect is significant, the result still might be regarded as reporting a mediation effect. This mediation effect shows that emotional collective work-identity and exhaustion were positively related *only* through psychological job demands (see “Design and data analyses” section above and MacKinnon et al., 2000, 2007; MacKinnon, 2008; Hayes, 2009; Zhao et al., 2010; Hayes and Rockwood, 2016 for this type of interpretation).

All this suggests that when the cognitive processes of personal, and emotional processes of collective, work bonding increases, teachers may experience greater psychological job demands, which in turn may increase their exhaustion.

**Figure 5:**
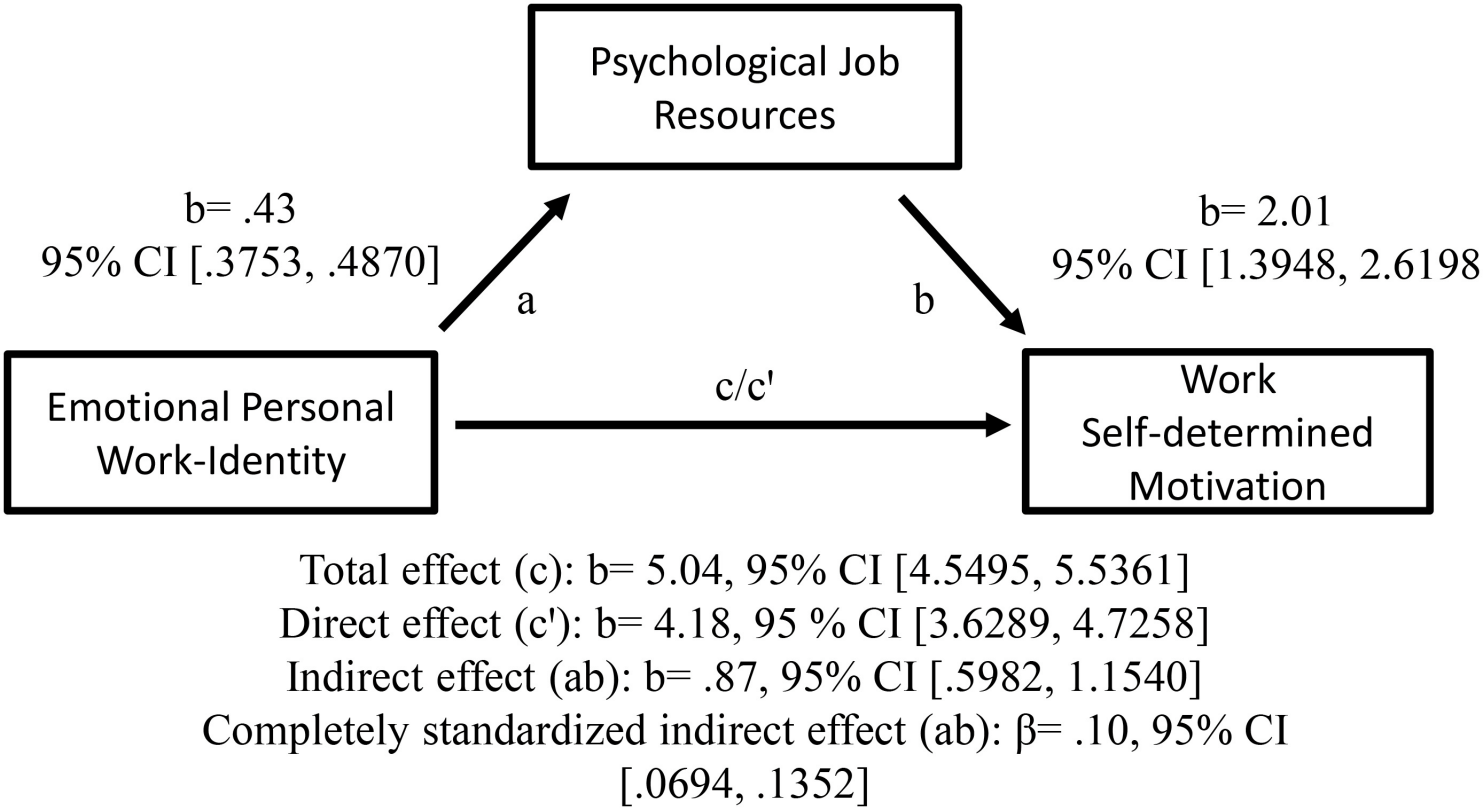
Emotional personal work-identity as a predictor of work self-determined motivation, mediated by psychological job resources. The confidence interval (CI) for the indirect—and completely standardized indirect effect is a BC bootstrapped CI based on 5,000 samples.

**Figure 6:**
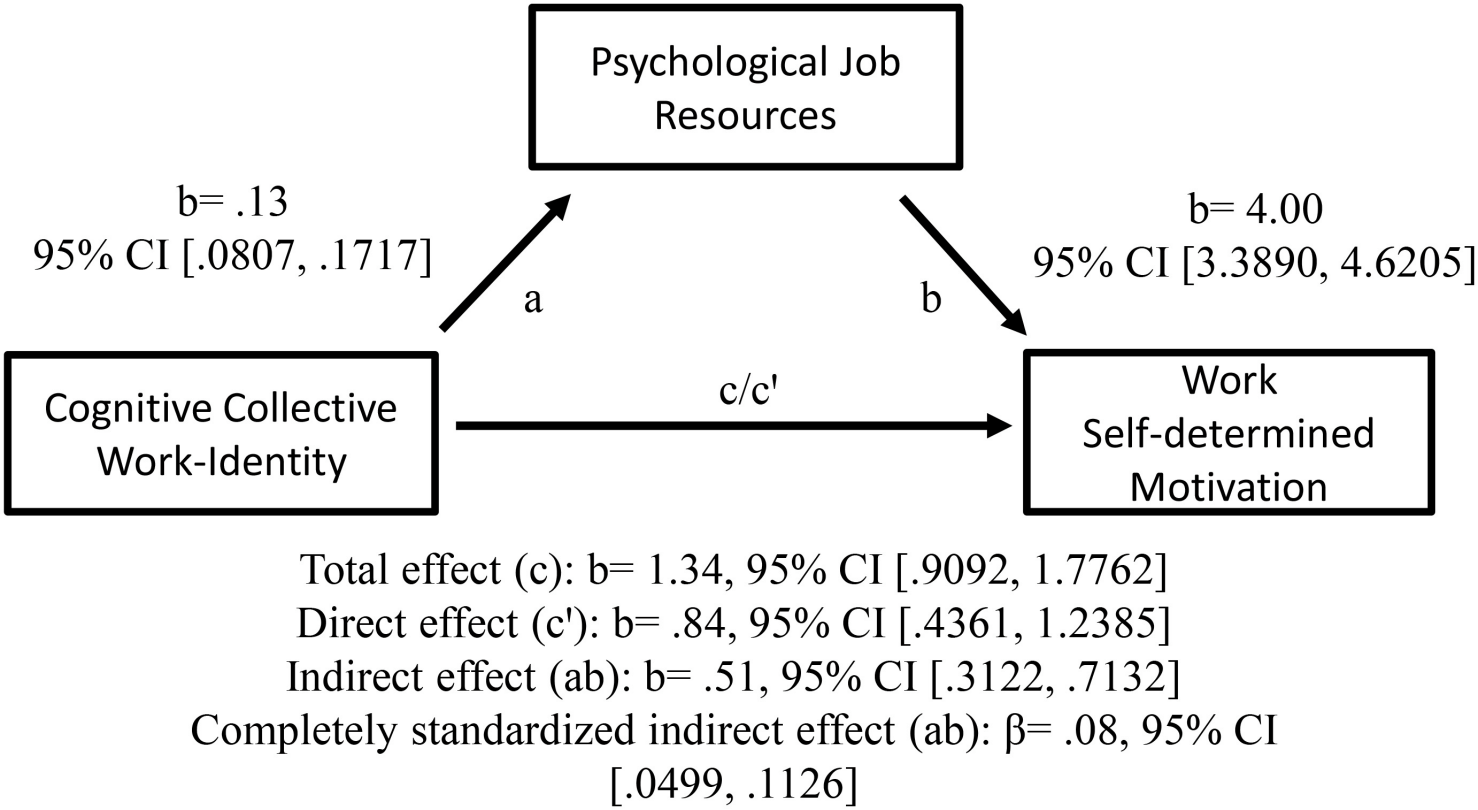
Cognitive collective work-identity as a predictor of work self-determined motivation, mediated by psychological job resources. The confidence interval (CI) for the indirect—and completely standardized indirect effect is a BC bootstrapped CI based on 5,000 samples.

As predicted, it was also shown that the positive relationships between teachers' emotional personal work-identity (H2a) and cognitive collective work-identity (H2b), respectively, and self-determined motivation at work were mediated by psychological job resources (in terms of educational inspirations); see Figures 5, 6 for details. Thus, teachers' emotional personal work-identity and cognitive collective work-identity, respectively, predicted work self-determined motivation indirectly via psychological job resources.

This suggests that when the emotional processes of personal and the cognitive processes of collective work bonding increase, teachers may experience greater psychological job resources, which in turn may increase their self-determined motivation at work.

## Discussion

The objectives of the present study were to investigate the mediating roles of teachers' psychological job demands and resources in the relationships between personal and collective work-identity, respectively, and exhaustion and self-determined work motivation.

The results obtained were in line with our predictions (see Figures 2A,B, and Hypotheses section) and previous findings (e.g., Hakanen et al., 2006, 2008; Klusmann et al., 2008; Hultell and Gustavsson, 2011; Badawy, 2015; Karatepe, 2015). That is: (1) Teachers' psychological job demands positively mediated the positive relationship between cognitive personal work-identity and exhaustion. Emotional collective work-identity and exhaustion were positively related *only* through the mediation of teachers' psychological job demands. (2) Teachers' psychological job resources positively mediated the positive relationship between emotional personal work-identity and cognitive collective work-identity, respectively, and self-determined motivation at work.

Accordingly, when (H1a) cognitive processes of coherence, correspondence, mental time, reflection and agency of personal work bonding increase, teachers may experience greater psychological job demands in terms of prosocial extra-role performance, such as “extra-role support given to the pupils; colleagues; students' parents” (Hakanen et al., 2006; Bolino et al., 2010). This, in turn, may increase their exhaustion (see van der Ploeg and Kleber, 2003; Janssen and Nijhuis, 2004; Bolino et al., 2010; Van de Ven et al., 2013).

Similarly, when (H1b) emotional processes of pride, esteem, and affective commitment of collective work bonding increase, teachers may also experience greater psychological job demands in terms of prosocial extra-role performance (see Hakanen et al., 2006; Bolino et al., 2010). This may, as above, increase their exhaustion (see van der Ploeg and Kleber, 2003; Janssen and Nijhuis, 2004; Bolino et al., 2010; Van de Ven et al., 2013).

Consequently, and in line with the Job Demands-Resources model (Demerouti et al., 2001; Hakanen et al., 2008), the present study showed that teachers' psychological job demands (operationalized as prosocial extra-role performance) may constitute an explaining factor (mediator) in the links of their *personal* work-related *thinking* and their *collective* work-related (organizational/school) *feeling*, respectively, and their experiences of exhaustion. Thus, and in contrast to some results showing prosocial extra-role performances to be positively related to mental health (Simbula and Guglielmi, 2013; Lam et al., 2016), our results are in line with findings indicating a “dark side” of this construct, in other words, suggesting a positive link with exhaustion (Bolino et al., 2004, 2013; Vigoda-Gadot, 2006, 2007; Bolino and Grant, 2016).

Furthermore, when (H2a) the emotional processes of attachment/belongingness/closeness of personal work bonding increase, teachers may experience stronger psychological job resources in terms of educational inspiration in their interplay with students and the overall educational work (see Bakker and Demerouti, 2008; Bakker and Bal, 2010; Choochom, 2016). This, in turn, may increase their self-determined motivation at work (see Vansteenkiste et al., 2007; Hakanen et al., 2008).

Similarly, when (H2b) cognitive processes of incorporation, identification and assimilation of collective work bonding increase, teachers may experience stronger psychological job resources in terms of educational inspiration in their interplay with students and the overall educational work (see Bakker and Demerouti, 2008; Bakker and Bal, 2010; Choochom, 2016). This may increase their self-determined motivation at work (see Vansteenkiste et al., 2007; Hakanen et al., 2008).

Consequently, and in line with the Job Demands-Resources model (Demerouti et al., 2001; Hakanen et al., 2008), the present study showed that teachers' psychological job resources (operationalized as educational inspiration) may constitute an explaining factor (mediator) in the links of their *personal* work-related *feeling* and their *collective* (organizational/school) work-related *thinking*, respectively, and their self-determined motivation at work. These results indicate that educational inspiration may constitute a psychological resource for teachers' self-determined motivation at work (Bakker and Demerouti, 2008; Miquelon and Vallerand, 2008, Vansteenkiste et al., 2007; Bakker and Bal, 2010; Choochom, 2016) because, as an intrinsic goal, it might facilitate basic needs of satisfaction of autonomy, belongingness and competence (Deci and Ryan, 2000; Hakanen et al., 2008; Ryan and Deci, 2008; Van Den Broeck et al., 2008). In addition, intrinsic goals might increase satisfaction and well-being (Ryan and Deci, 2000b; Björklund et al., 2013), while extrinsic motivation may relate positively to anxiety (Gillet et al., 2016).

In sum, from a theoretical and an applied perspective, our results suggest that teachers might be *disadvantaged* by stronger personal work-related thinking, organizational/school-related feelings and prosocial extra-role performance since these factors, to some extent, were positively associated with their exhaustion. In other words, teachers' prosocial extra-role performance (a psychological job *demand*) may play a positive mediating role in the relationships between teachers' cognitive personal and emotional collective work-related bonding, respectively, and exhaustion.

Furthermore, teachers might find *advantage* in stronger personal work-related feelings, organizational/school-related thinking and educational inspiration since these factors, to some extent, might enhance their self-determined motivation at work. Consequently, teachers' educational inspiration (a psychological job *resource*) may play a positive mediating role in the relationships between teachers' emotional personal and cognitive collective work-related bonding, respectively, and self-determined motivation at work.

By focusing on personal and social identity perspectives, the present study showed that work conditions as defined by the Job Demand-Resource model (Demerouti et al., 2001; Demerouti and Bakker, 2011) might account for relations between the individual employee and adverse mental health and work motivation. Thus, the present study contributes to the understanding of how teachers' personal and collective work-identity, exhaustion and work motivation, may be mediated by their psychological job demands and resources.

It may be relevant to discuss some practical implications of the present study. Teachers' educational inspiration should be promoted since such psychological job resources may positively mediate the relationships between personal work-related feeling (i.e., concerning teaching) and organizational/school-related thinking, respectively, and self-determined motivation at work. Teachers' prosocial extra-role performances should be reduced since they may mediate positively the relationships between teachers' cognitive personal and emotional collective work-related bonding, respectively, and their exhaustion problems.

Promotion of teachers' educational inspiration and reduction of their prosocial extra-role performances might be implemented for example, by clearer cut and explicit work descriptions and expectations of in-role vs. extra-role performances within the national educational system programs. Here, increased job autonomy of teachers should also be promoted since it might buffer the negative effects of prosocial extra-role performances (see Vigoda-Gadot, 2007; Klusmann et al., 2008; Somech, 2016).

Reduction of teachers' work-related thinking as well as promotion of their work-related feeling and school-related (organizational) thinking by mental training programs may reduce their exhaustion problems and increase work motivation (see van Dierendonck et al., 1998; van der Klink et al., 2001; Lloyd et al., 2016).

Such improvement of teachers' psychosocial work environment may also increase their sense of self-efficacy and job control, thereby decreasing burnout and exhaustion problems (see Zołnierczyk-Zreda, 2005; Skaalvik and Skaalvik, 2007; Siu et al., 2014). Finally, a decrease in teachers' exhaustion problems is of importance due to the high prevalence of burnout and exhaustion problems (see van Dierendonck et al., 1998; Hallsten et al., 2002; Hakanen et al., 2006; Schaufeli and Taris, 2014; van Wingerden et al., 2016). Teachers' stronger educational inspiration and self-determined work motivation and lesser degree of prosocial extra-role performances and exhaustion might also increase their ability to perform adequate teaching and achieving educational goals. This in turn may mobilize interest, energy, excitement, and performance among the pupils (see Patrick et al., 2000; Bakker, 2005; Hakanen et al., 2006).

## Limitations

Finally, it is appropriate to mention four limitations of this study. First, as the results obtained are based on cross-sectional data; thus, lacking random assignment, it is not possible to draw absolute conclusions about causation. Second, we did not include any specific school-related and educational variables in the analyses because the aim was to investigate the general relationships between the phenomena involved. Third, the operationalization of psychological job demands and resources was limited to teachers' prosocial extra-role performance and educational inspiration, respectively. Fourth, due to the study's ethical considerations we could not collect any data on teachers' school affiliations. Therefore, no intra-class-correlations (ICC) were calculated. However, we estimate that our sample includes about 300 to 500 different schools. Given the large number of schools the number of participants per school was so low that no meaningful ICC-analysis on school level was possible to calculate.

## Conclusions

The present study contributes to the understanding of teachers' work-identity and psychosocial working conditions in relation to their exhaustion problems and motivation at work in that: (1) teachers' personal work-related *thinking* and collective work-related *feeling* associates positively with their exhaustion problems indirectly via psychological job *demands* (prosocial extra-role performances); (2) teachers' personal work-related *feeling* and collective work-related *thinking* associates positively with their work motivation indirectly via psychological job *resources* (educational inspiration): (3) teacher's psychological job demands and resources might to some extent constitute *explaining factors* between the individual teacher (work-identity) and exhaustion problems and work motivation, respectively.

## Data Availability Statement

All datasets generated for this study are included in the article/supplementary material.

## Ethics Statement

The studies involving human participants were reviewed and approved by Swedish Regional Ethical Committee of Uppsala University (Dnr 2015/423). The patients/participants provided their written informed consent to participate in this study.

## Author Contributions

ON: substantial contributions to the conception and design of the work, data collection, manuscript writing, analysis and interpretation of data for the work, and drafting the work critically for important intellectual content. IK: substantial contributions to the conception and design of the work, manuscript writing, interpretation of data for the work, and drafting the work critically for important intellectual content. FS and JW: manuscript writing, interpretation of data for the work, and drafting the work critically for important intellectual content. All authors contributed to the article and approved the submitted version.

## Conflict of Interest

The authors declare that the research was conducted in the absence of any commercial or financial relationships that could be construed as a potential conflict of interest.
